# The cost and value of cancer drugs – are new innovations outpacing our ability to pay?

**DOI:** 10.1186/s13584-016-0097-0

**Published:** 2016-09-22

**Authors:** Daniel A. Goldstein, Salomon M. Stemmer, Noa Gordon

**Affiliations:** 1 Davidoff Cancer Center, Rabin Medical Center, Petach Tikvah, Israel; 2 Winship Cancer Institute, Emory University, Atlanta, GA USA; 3 Sackler School of Medicine, Tel Aviv University, Tel Aviv, Israel; 4 Ben Gurion University of the Negev, Beer Sheva, Israel

## Abstract

Cancer drug expenditures have been increasing significantly in countries around the world. A recent paper in the IJHPR provides new knowledge and insights into this global phenomenon by analyzing how it is playing out in an Israeli health plan with over two million members, whose state-of-the-art information systems provide an opportunity to explore these changes in a comprehensive, detailed and reliable manner. There is a wide variation in both the cost-effectiveness and the budget impact of individual drugs. These issues also vary when analyzing drugs in other countries due to differential pricing mechanisms. In addition to drug expenditure, the overall cost of cancer care is increasing, partly due to expenditures on non-pharmacologic treatments and diagnostic testing. With the arrival of new therapies, the future of cancer care is exciting. However, there will be many challenges ahead with regard to the ability to pay for such innovations. In this commentary we discuss the current problems and anticipate the future challenges.

## Background

Cancer drug costs have become a major concern in policy discussions and in the mainstream media. The costs of cancer care are not only challenges for governments, and insurance companies, but they are increasingly placing a financial burden on patients. Lomnicky et al. have demonstrated the significant increase in expenditure on cancer drugs by Maccabi Healthcare Services, an Israeli health plan which insures more than two million people. This health plan has state-of-the-art information systems which provide a unique opportunity to explore these changes in a comprehensive, detailed and reliable manner, for a large and relatively stable population over a long period of time. Conversely they have demonstrated that expenditure on cardiac drugs has decreased while drug expenditure for non cardiac or non-cancerous diseases has remained relatively constant [1]. These data provoke many thoughts and questions regarding cancer drug costs. Why is the expenditure on cancer drugs increasing? Are we getting value for money? How significant is this increase when considering the whole healthcare budget? How does Israel fare compared to other countries in the world regarding cancer drug costs? What does the future hold, and will this expenditure continue to grow? Will the movement towards personalized therapy, using genetic testing with targeted therapy lead to an increase or decrease in expenditure? In this commentary we will attempt to answer these questions.

## Cost versus value

Understanding costs is important especially when working within a fixed budget. However, perhaps the more important question is cost-effectiveness or value. Paying 12,000 shekels (approximately $3000) for a month of drug treatment may sound a lot, however if this cures a deadly disease, we can consider it to be highly cost-effective. On the other hand, paying the same amount for a drug that increases life expectancy by just 6 to 12 weeks is totally different, and would not be considered cost-effective by most conventional thresholds. To explore this issue, it is worth considering diseases aside from cancer. Although the recent introduction of drugs to treat hepatitis C came at a high price tag, the efficacy is tremendous, with the potential to cure the disease. There are significant differences in prices around the world, but in the US, a 12 week course of sofosbuvir costs approximately $65,000 [2]. Despite such high prices in the US, such drugs have been demonstrated to be cost-effective due to the significant benefit to patients [3].

In contrast, bevacizumab is used in colorectal cancer and increases life expectancy by only about 6 weeks. In Israel this costs approximately 12,000 shekels per month, while in the United States it costs approximately $5300 per month. Based on prices in the US, this treatment is not considered cost-effective by all conventional thresholds – although its use is widespread [4]. Despite many advances in the management of cancer, the drugs required come at high prices, and the duration of therapy is often extensive. For example, the development of pertuzumab has been hailed as a major achievement in the ability to treat *HER2* positive breast cancer. However, given the high cost (approx. $215,000 per patient [5]), it has also been demonstrated not to be cost-effective in metastatic disease, based on US prices [5]. Many drugs used in the metastatic setting (stage 4/advanced) may have low cost-effectiveness given that they extend life by just a matter of weeks. However, drugs used in the adjuvant setting (early stage/curative), may be much more cost-effective given the potential for cure. Such drugs are used to decrease the chance of disease recurrence following surgery, and thus often convey a significant clinical benefit.

## The patient’s perspective or the healthcare system’s perspective?

When considering healthcare costs, traditionally the area of greatest focus has been the perspective of the institutional payer such as governments or insurance companies. However we are increasingly learning that the patients’ perspective is important to consider with regard to the financial burden that they incur related to treatment. Recent research from Washington State has demonstrated that people with a diagnosis of cancer have a 2.7 times higher risk of bankruptcy, than people without cancer [6]. However, the story does not stop there. Patients who declared bankruptcy had a 79 % greater mortality risk than patients who had not declared bankruptcy, suggesting that significant financial distress has a negative impact on health outcomes [7]. These financial problems in the United States are largely related to cost-sharing, with high deductibles and copays. One may argue that this is not a concern within the public system in Israel and other countries with universal health care coverage. However, we would propose that many patients in Israel take on considerable financial burden as a result of payments for second opinions, additional tests, and drugs not included within the basket of services. So while the cost-sharing is not a major problem for drugs in the basket, it is a problem when patients decide to do additional tests and treatments not included in the basket.

Even more significantly, growing expenditures on cancer drugs can crowd out funding for other important medications and/or other treatments. This is most obvious in countries like Israel that explicitly set aside a certain amount of money each year for the funding of new technologies [8], forcing explicit prioritization and head-to-head comparisons of candidates for addition to the benefits package – taking into account both costs and benefits. But even in countries without annual budget caps for new technologies, overall health care resources are typically limited and budget constrained, so that increased expenditures on cancer drugs can ultimately come at the expense of treatment for a wide range of significant threats to population health, including heart disease, mental health, arthritis, and dementia. While this does not imply that increased spending on cancer drugs is necessarily a bad thing, it does suggest that the health benefits of such increases need to be weighed against the benefits to be gained from alternative uses of the funds.

## Global variations in drug prices

Drug prices and accessibility vary significantly around the world. While some drugs within Europe have equivalent prices in different countries due to “reference pricing” [9], many drugs carry higher price tags in the United States [10, 11]. However, understanding the actual amount of money that changes hands is a major challenge. Many different programs of negotiations exist between countries and within countries, including programs for risk sharing, discounting and rebates. The details of such programs are often a high level secret, thus reducing transparency and the ability to truly understand value.

## Are drugs the only topic of concern in the cost of cancer care?

While the high cost of cancer drugs has received a lot of attention recently, it is important to put this expenditure into perspective. In the United States, drug spending constitutes approximately 20 % of total cancer spending for commercially insured individuals [12]. However it is important to recognize that cancer drug spending is a growing sector within health spending, and thus warrants scrutiny. It is also important to consider that there is great expenditure in other fields of cancer care, including radiology, radiation oncology, surgery, molecular diagnostics and costly interventions at the end of life. Within these fields and others, there are definitely areas of low value care, where efficiency could be improved. We believe that it is important to maintain a focus on high quality, high value care throughout all fields of medicine.

## The future: targeted therapy, immunotherapy and biosimilars

Lomnicky et al. have demonstrated that cancer drug expenditure has increased in the previous decade, but what can we expect in the next decade? The world of cancer care is entering an exciting era, with the arrival of immunotherapy and the further development of treatment tailored specifically to the genetic makeup of an individual’s tumor. Drugs such as nivolumab and pembrolizumab inhibit the programmed cell death protein 1 (PD-1) on tumors and thus “remove the brakes” for an individual’s immune system to fight against a tumor. Such drugs have already gained approval in the management of melanoma, lung and kidney cancer, with more approvals expected in the coming months and years [13–16]. These drugs can also be paired with cytotoxic T-lymphocyte-associated protein 4 (CTLA4) inhibitors such as ipilumumab, thus stimulating the immune system by two separate mechanisms. While many patients do not gain any benefit from such drugs, a small proportion may gain durable responses, with the potential for major survival benefits, never before seen in these and other malignancies. Targeted therapy is also holding great promise and lung cancer therapy is the poster child for this field. Patients with this disease now undergo genetic testing of their tumors to evaluate for *EGFR* mutations or *ALK* translocations in order to treat with either class of drugs for both mutations and alterations [17, 18]. Despite subsequent resistance that usually develops, further 2^nd^ generation tyrosine kinase inhibitors have been developed to target the resistant mutations. These include ceritinib and osimertinib [19, 20]. It is expected that new targeted therapies will be developed in the coming years in all fields of cancer. Although the advances in immunotherapy and targeted therapy are exciting, the costs will pose a major challenge, particularly as combination therapies are introduced. It is likely that risk-sharing will be required in order to fund such interventions.

After patent expiry we have traditionally seen major decreases in drug prices following the arrival of generic drugs. This has been of major importance for the budgets of healthcare payers. However a major challenge is to be expected in the coming years related to biologic therapies. Many new cancer drugs are biological agents, and only biologically similar agents can be produced following patent expiration. Regulatory agencies have required that such biosimilar drugs go through basic testing to demonstrate pharmacological equivalency, however stringent clinical trials are not required. Despite this, however, the cost of development of such biosimilar drugs is considered to be higher than the development of generic drugs. As a result, the drug price reductions at patent expiration are not expected to be significantly less than those for generic drugs, however this may also be simply because of a lack of significant competition. Generic drug prices have traditionally fallen to around 10 % of their original price. However biosimilar drug prices are projected initially to fall only to approximately 80 %. As a result, it is expected that drug budgets may face significant challenges in the future.

## Conclusions

There is much excitement about new developments in the field of cancer care. While some of these developments hold great promise, many new drugs confer only a minimal level of clinical benefit. Scientists, society and policy makers will be increasingly challenged with decisions of how to spend finite resources. Clear definitions and understanding of both cost-effectiveness and budget impact are essential in order to make high value coverage decisions.
